# Preparation and Characterization of Antibacterial Cellulose/Chitosan Nanofiltration Membranes

**DOI:** 10.3390/polym9040116

**Published:** 2017-03-23

**Authors:** Rengui Weng, Lihui Chen, Shan Lin, Hui Zhang, Hui Wu, Kai Liu, Shilin Cao, Liulian Huang

**Affiliations:** 1 College of Material Engineering, Fujian Agriculture and Forestry University, Fuzhou 350002, China; wengrengui109@126.com (R.W.); ls99036@163.com (S.L.); zhangh10@163.com (H.Z.); wuhuifafu@163.com (H.W.); liuk1103@163.com (K.L.); fafucsl@fafu.edu.cn (S.C.); hll65212@163.com (L.H.); 2 College of Ecological Environment and Urban Construction, Fujian University of Technology, Fuzhou 350118, China

**Keywords:** cellulose, chitosan, antibacterial activity, nanofiltration membranes

## Abstract

Presently, most nanofiltration membranes are prepared with non-biodegradable petrochemical materials. This process is harmful to the ecosystem and consumes a large amount of non-renewable energy. In this study, biodegradable and biocompatible antibacterial cellulose/chitosan nanofiltration membranes (BC/CS-NFMs) were fabricated and characterized for their mechanical strength, antimicrobial activity, salt and dye filtration performance, and polyethylene glycol (PEG) retention using Thermal gravimetric analysis (TGA), Field emission scanning electron microscopy(FE-SEM), Fourier transform infrared spectroscopy(FT-IR), and X-ray diffraction (XRD). The BC/CS-NFMs were obtained by the hydrolysis and carboxymethylation of dense cellulose/chitosan membranes (BC/CSMs). The tensile strength of the BC/CS-NFMs decreased as the chitosan content increased. In addition, the thermal stability and antibacterial ability of the BC/CS-NFMs improved. The pore size is less than 1 nm, and a spongy, layered structure is observed in the cross-sectional FE-SEM images. FT-IR analysis shows that a part of the hydroxyl in cellulose transforms to carboxymethyl during the hydrolysis and carboxymethylation of the BC/CSMs. No obvious changes can be observed in the cellulose and chitosan after the blend membrane formation from the XRD measurements. Based on the experimental results on the permeation and rejection of BC/CS-NFMs, different proportions of cellulose and chitosan nanofiltration membranes almost did not affect the water flux and rejection rate. The BC/CS-NFMs showed better water flux and a higher rejection rate in aqueous dye-salt solutions.

## 1. Introduction

Nanofiltration (NF) is a pressure-driven and low energy consumption membrane separation technology that has been significantly developed in the last decade [1]. Generally, a nanofiltration membrane can cut off by molecular weights ranging from 200 to 1000 Da and pore sizes with a diameter of approximately 0.5–2.0 nm [2]. Due to its ability to separate low-molecular-weight organic species and metal ions, it has become an important separation and purification technique in the water and gas industries, wastewater reclamation, separation of substances, drinking water purification, sea water desalination, industrial fluids treatment, and so on [3,4,5,6,7,8,9]. Currently, most nanofiltration membranes are prepared with non-biodegradable petrochemical materials; polyethersulfone (PES) and polyvinylidene fluoride (PVDF) membranes are mostly sold as microfiltration or ultrafiltration membranes, and they usually require further modifications to behave like NF membranes. PES nanofiltration membranes are prepared by blending negatively charged surface modifying macromolecules (cSMM) in the spinning formulation through the phase-inversion technique [10]. Polysulfone nanofiltration hollow fiber membranes are fabricated through a UV-photografting process in their outer surface [11]. Prepared PVDF nanofiltration membranes were fabricated through an interfacial polymerization. The membranes were prepared on the support of PVDF ultrafiltration membranes with piperazine (PIP) as the aqueous monomer and trimesoylchloride (TMC) as the organic monomer [12]. More recently, a composite capillary membrane for solvent resistant nanofiltration was developed through coating a selective poly(dimethylsiloxane) (PDMS) top layer on an α-alumina support [13]. However, these materials lead to energy waste and destruction of the ecological environment. So developing an environmentally friendly biodegradable membrane has great significance for the ecological environment. 

It is vital to find ideal materials to replace petrochemical materials for membrane production. Cellulose is one of the most abundant renewable, biodegradable, and inexpensive organic materials, and it is considered to be environmentally friendly and a biocompatible product. It has been used to prepare separation membranes, but the cellulose membrane has no antimicrobial properties [14]. The cellulose membrane is susceptible to microbial erosion, which influences the stability and service life of membrane. So it is necessary to make the cellulose membrane with antimicrobial properties. Chitosan is a biodegradable material with antimicrobial properties [15,16]. Thus, the key issue is to use a low-toxicity and recyclable solvent to simultaneously dissolve both types of materials.

To date, previous cellulose-dissolving studies have observed that these solvents include trifluoroacetic acid, Acetobacter xylinum, NaOH/thiourea, ionic liquids, and ZnCl_2_·3H_2_O [17,18,19,20]. However, those traditional dissolution processes face many challenges because of high cost and the toxicity or difficulty of solvent recovery. Therefore, to make full use of cellulose and chitosan resources, it is necessary to develop a green cellulose/chitosan dissolution method. *N*-methylmorpholine-*N*-oxide (NMMO) is indisputably considered to be the most promising organic solvent because it is non-toxic, non-corrosive, non-volatile, easily recyclable, and environmentally friendly [21].

The ability of NMMO to directly dissolve cellulose has been known for many years. The chemical structure of the chitosan backbone is relatively similar to that of cellulose, other than the functional groups connected to the second carbon in the repeating units that differ. The ability of NMMO to directly dissolve chitosan under vacuum conditions has been previously reported. In recent years, the use of NMMO as a cosolvent mainly focused on cellulose/chitosan ultrafiltration membranes, blend membranes, microspheres, etc. [22,23]. High-flux and anti-fouling cellulose nanofiltration membranes were prepared with an ionic liquid as the solvent [24]. However, the membranes have no antimicrobial properties, and they must be kept in water to prevent curling. To date, there have been no reports on cellulose/chitosan nanofiltration membranes obtained by hydrolysis and carboxymethylation. 

The main objective of this study is to establish an environmentally friendly and low cost method to prepare nanofiltration membranes from regenerated cellulose with antimicrobial properties. In this study, cellulose and chitosan were dissolved in NMMO; membranes were produced using a coating machine; cellulose/chitosan membranes were obtained by rinsing with water and drying; and cellulose/chitosan nanofiltration membranes were obtained by hydrolysis and carboxymethyl modification. This research explores the chemical and physical properties, antibacterial properties, cut-off performance, permeation, and rejection properties of nanofiltration membranes.

## 2. Experimental Section

### 2.1. Materials

Bamboo cellulose (BC) with a polymerization degree of 650 was kindly provided by Sichuan Tianzhu Bamboo Resources Development Co. Ltd. (Yibin, China). The BC was dried overnight at 60 °C prior to dissolution. Chitosan (CS, *M*_w_ = 2 × 10^5^ Da, degree of deacetylation = 90%) was purchased from Golden-Shell Biochemical Co., Ltd. (Yuhuan, China). 10 g of chitosan was added to 3 wt % acetic acid to obtain an aqueous chitosan acetic acid solution. 5 wt % of NaOH was dropped into the aqueous chitosan acetic acid solution. The chitosan was solidified and regenerated into particles. The solid particles were washed with water repeatedly, dried at 105 °C, and crushed through a 120-mesh sieve to obtain powder chitosan [25]. *N*-methylmorpholine-*N*-oxide (NMMO) (Analytical reagent >97%) was obtained from Tianjin Hainachuan Science and Technology Development Co., Ltd. (Tianjin, China), Polyethylene glycol (PEG) (*M*_w_ = 400, 600, 800, 1000, and 2000 Da), propyl gallate, methyl orange (327.33 g/mol), and methyl blue (799.80 g/mol) dyes were purchased from Aladdin Chemical Regent Co., Ltd., Shanghai, China. The water used in this experiment was de-ionized water.

### 2.2. Preparation of Cellulose/Chitosan Nanofiltration Membranes

Four formulations with different weight proportions (BC/CS = 4:1, 6:1, 8:1, 10:1) were used. The BC/CS solution was prepared by dissolving a particular amount of cellulose and chitosan powder in 86.7% NMMO aqueous solution in a flask, and the mixture was heated at 110 °C in a pumped vacuum and stirred until the BC/CS samples were completely dissolved. The BC/CS/NMMO solution with a BC/CS concentration of 6 wt % was obtained. The PET non-woven fabric was fixed onto a glass plate of coater (GBC-A4, Gwangju Institute of Science and Technology, Gwangju, Korea). The solution was poured onto the non-woven fabric, and the roll was moved with a speed of 20 mm/s. The blend membrane was immersed in a water coagulation bath at room temperature. Then, the blend membranes were washed with water to remove the residual solvent. Finally, the blend membranes were air-dried at room temperature to obtain dense cellulose/chitosan membranes (BC/CSMs) [26,27,28].

**Figure 1:**
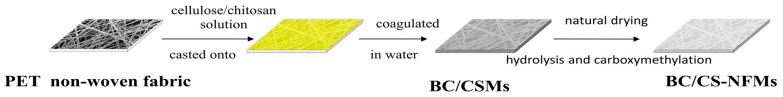
The scheme for fabricating cellulose/chitosan nanofiltration membranes.

To obtain antibacterial cellulose/chitosan nanofiltration membranes (BC/CS-NFMs), the BC/CSMs were modified by hydrolysis and carboxymethylation. First, hydrolysis was carried out by treating the BC/CSMs at 30 °C in 1 mol/L NaOH solution for 30 min. Afterwards, the treated BC/CSMs were washed with water. Then, carboxymethylation was performed by treating the hydrolyzed BC/CSMs above at 60 °C in 3.0 wt/v % chloroacetic acid and NaOH (*n*_NaOH_:*n*_Chloroacetic acid_ = 2.5:1) for 60 min. The carboxymethylated membranes were then washed with water [29]. This process is illustrated in Figure 1.

### 2.3. Characterization of Cellulose/Chitosan Nanofiltration Membranes

The cellulose powder, chitosan powder, cellulose membrane (BCM), BC/CSMs, and BC/CS-NFMs were tested by Fourier transform infrared spectroscopy (FT-IR). The spectra with a wavenumber ranging from 4000 to 400 cm^−1^ were recorded on a Fourier transform Infrared spectrometer (Thermo Nicolet 380, Thermo Fisher Scientific, Waltham, MA, USA) by the KBr-disk method.

The surface and cross-sectional morphology of the BC/CS-NFMs and BC/CSMs were investigated with a FEI Nova NanoSEM450 field emission scanning electron microscopy (FE-SEM), (FEI, Hillsboro, OR, USA). The samples were freeze-fractured in liquid nitrogen, and then sputtered with gold in a sputtering device.

According to the ASTM D 882-02 standard (ASTM, 2002), the membranes were cut into strip-shaped specimens with a width of 10 mm and a length of 50 mm. The specimens were measured on a universal tensile tester (ZQS13-300, Sichuan Changjiang Papermaking Machine Co. Ltd., Luzhou, China), with a stretch speed of 30 mm/min and a clip distance of 40 mm. The tensile strength of a specimen was determined from the average value of 10 similar specimens.

The crystal structures were analyzed by X-ray diffraction (XRD). The XRD measurements were carried out in reflection mode on a MiniFlex2 XRD diffractometer (Rigaku, Tokyo, Japan) with a Cu K-radiation of 1.54 Å at 40 kV and 30 mA. The patterns were obtained in the 2θ range from 5° to 80°.

The relationship between the material mass loss and the change of temperature was determined with a TG-DTA instrument (Netzsch STA 449 F3, Netzsch, Deseb, Germany) at a heating rate of 10 °C/min under nitrogen with a flow rate of 20 mL/min. Each sample was weighed as approximately 2 to 3 mg as a standard and heated from 30 to 500 °C.

### 2.4. Antibacterial Assessment

*Escherichia coli* (ATCC25922) was selected as the bacteria in the tests. The antimicrobial activities of BCM and BC/CS-NFMs were tested by the disc diffusion method. Peptone (10 g), yeast powder (5 g), and NaCl (5 g) were mixed in 1000 mL water as a nutrient medium. The pH of this culture was adjusted to approximately 7.2 with NaOH, and then the culture was added to 2 wt % agar in 500 mL nutrient medium under stirring. Finally, the nutrient medium and nutrient agar medium were sterilized in respective conical flasks at a pressure of 0.1 MPa for 30 min. *Escherichia coli* was moved into the sterilized nutrient medium, oscillated for a few minutes, and maintained at a constant temperature of 37 °C for 24 h. When the medium was turbid, it showed the strain’s activation success. Suspensions (0.1 mL) of *Escherichia coli* were transferred to 500 mL of the sterilized nutrient agar medium [30,31]. The membranes were placed on the *Escherichia coli* agar plate and incubated at 37 °C for 24 h. Then, the inhibition zone was monitored.

### 2.5. Cross-Flow Permeation Tests

**Figure 2:**
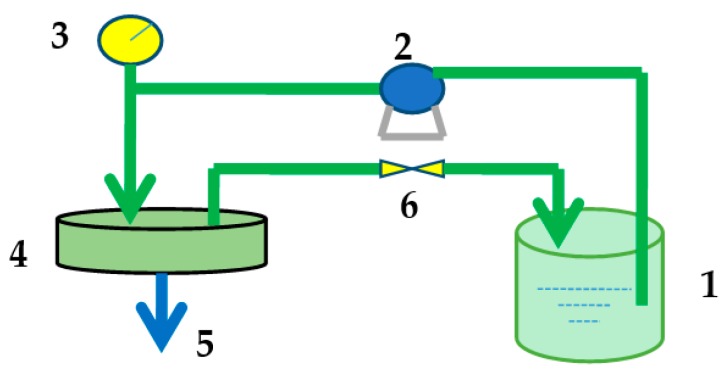
Membrane performance evaluation instrument; 1: Feed tank; 2: Pump; 3: Pressure gauge; 4: Membrane cell; 5: Permeate end; 6: Valve.

Permeations tests for dye and salt solutions were performed at a designed pressure using a flat-sheet cross-flow permeation test cell with a membrane area of 50.24 cm^2^. The equipment used for to evaluate the membrane performance is shown in Figure 2. All the membranes loaded in the equipment were pressurized with water under 0.5 MPa for at least 30 min before testing to obtain a stable membrane water flux. The temperature of the feed tank was constant by using the water-bath.

The permeation flux of the membrane was calculated using the following equation:

(1) J=V/(A×t)

where *J* is the permeation flux (L/m^2^·h), *V* is the permeate volume (L), *A* is the membrane area (m^2^), and *t* is the permeation time (h).

A conductivity meter (STARTER 3100C, Ohaus, Parsippany, NJ, USA) was used to determine the solute concentrations in the permeate and feed. The dye concentration was measured using a UV-visible spectrophotometer (Agilent 8453, Richardson, TX, USA) at the maximal absorption wavelength of each organic dye. The membrane rejection rate (*R*) of the dye or salt was calculated as follows:

(2) $$R=100\%\times (C_{f}-C_{p})/C_{f}$$

where *R* is the rejection rate (%), *C*_f_ is the feed concentration (mg/L), and *C*_p_ is the permeate concentration (mg/L). All cross-flow permeation experiments were conducted at room temperature.

### 2.6. Characterization of Molecular Weight Cut-Off and Mean Pore Size

The molecular weight cut-off (MWCO) refers to the molecular weight of the smallest solute that can be intercepted by a membrane. By measuring the retention rate of solutes of different molecular weights (usually PEG), one can obtain the relationship curve between the retention rate of the membrane and the molecular weight of the solute. Usually, the molecular weight of the solute corresponding to a retention rate of 90% on the retention curve is defined as the MWCO of a membrane [32,33].

The experiment measures the retention performance of the solution to characterize the MWCO of the membrane by measuring PEGs of different molecular weights (*M*_w_ = 400, 600, 800, 1000, and 2000 Da). The concentration of PEG was determined by employing the chromogenic reaction method between PEG and iodine, which was determined from the UV-Vis spectrophotometry.

The following holds true, according to the Stokes-Einstein equation [34]:

(3) $$r=16.73\times 10^{-3}\times M_{w}{}^{0.557}$$

where *r* is the Stokes Radius (nm), and *M*_w_ is the molecular weight of PEG (Da).

## 3. Results and Discussion

### 3.1. Mechanical Strength Analysis of Cellulose/Chitosan Nanofiltration Membranes

**Figure 3:**
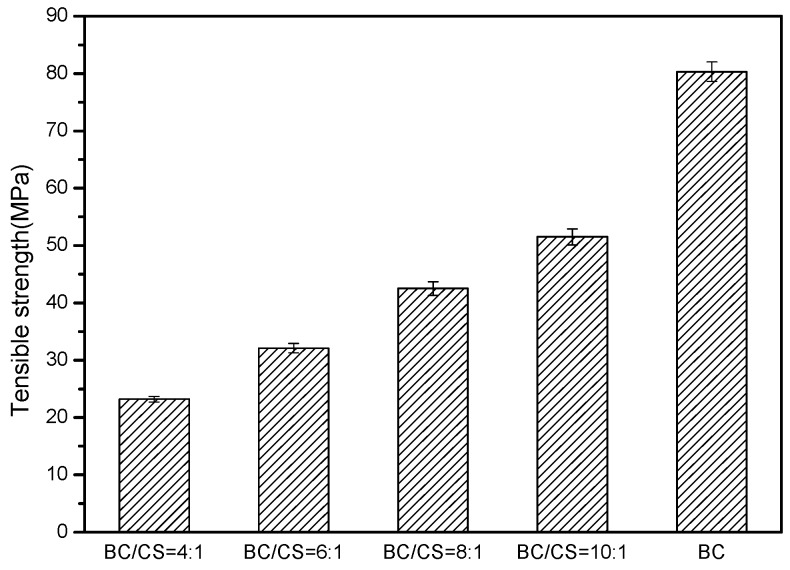
The tensile strength of cellulose/chitosan nanofiltration membranes.

Figure 3 shows the tensile strength of the blend nanofiltration membranes with different proportions of cellulose and chitosan. The tensile strength of each specimen was determined from the average value of 10 similar specimens. Obviously, the tensile strength of the pure cellulose nanofiltration membrane was the best, with a value of 80.3 MPa. By contrast, the tensile strength of the blend nanofiltration membranes decreased as the chitosan content increased, which suggests that the tensile strength of the blend nanofiltration membranes is dominated by cellulose. This is mainly attributed to the low bonding strength of chitosan itself, although cellulose could successfully combine with it.

### 3.2. TGA Analysis of Cellulose/Chitosan Nanofiltration Membranes

**Figure 4:**
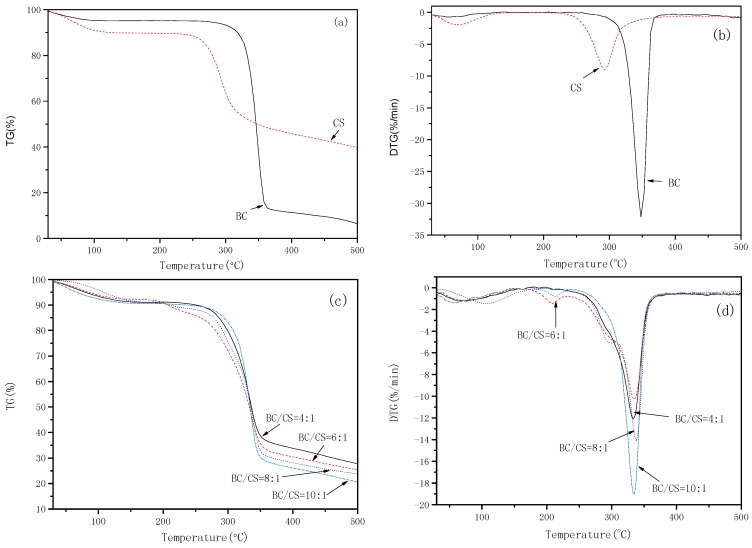
TG and DTG analysis results of cellulose and chitosan membranes (**a**,**b**); and blend membranes (**c**,**d**).

Figure 4 and Table 1 show the Thermogravimetry (TG) and Derivative thermogravimetry (DTG) curves of the cellulose, chitosan, and cellulose/chitosan nanofiltration membranes (BC/CS = 4:1, 6:1, 8:1, 10:1). The results indicated that the thermal stabilities of the cellulose membranes were superior to that of the chitosan membranes. The starting degradation temperature of the blend membranes decreased with the increase of the cellulose content, but only a slight change was observed for the four values. The same result was observed at the maximum degradation rate. This phenomenon may be mainly attributed to the similar chemical structure of cellulose and chitosan. Apparently, the residual quantities of the blend membranes increase as the chitosan content increases at 500 °C. This behavior may be explained by the interaction between the hydroxyl groups of the cellulose and the amino groups of the chitosan, which might disrupt the crystalline structures of the cellulose and chitosan.

### 3.3. Antimicrobial Activity Analysis of Cellulose/Chitosan Nanofiltration Membranes

**Figure 5:**
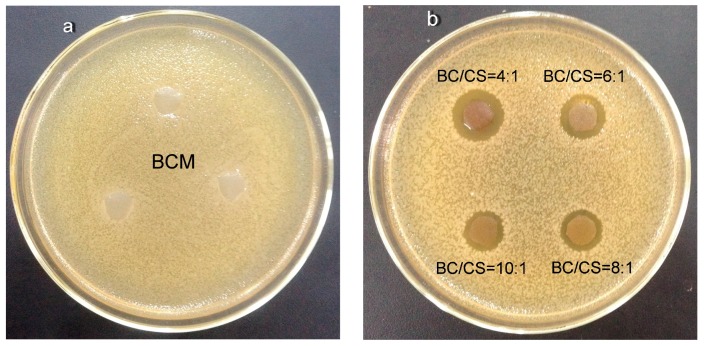
The photographs of BCM and BC/CS blend membranes obtained from the halo zone test. (**a**) The halo zone test of BCM; (**b**) The halo zone test of BC/CS blend membranes.

The inhibition zone test is conducted to assess the antibacterial ability of membranes by measuring the size of the halo zone and to determine whether membranes have antibacterial agents. The antibacterial ability of the BCM and BC/CS blend membranes was tested by *Escherichia coli* based on a disc diffusion method. Figure 5a shows that an inhibition zone does not appear around the BCM, while inhibition zones appear around the BC/CS blend membranes. This indicates that the BC/CS blend membranes contain antibacterial agents.

From Figure 5b, the inhibition zone grew larger as the chitosan content increased. The results illustrate that chitosan has antibacterial activity. The mechanism of the antimicrobial activity involves the interaction of the positively charged chitosan molecules with the negatively charged bacterial cell membranes, which leads to the loss of the membrane permeability and leakage of intracellular components, and then the killing of the *Escherichia coli*.

### 3.4. Morphology Analysis of Cellulose/Chitosan Nanofiltration Membranes

**Figure 6:**
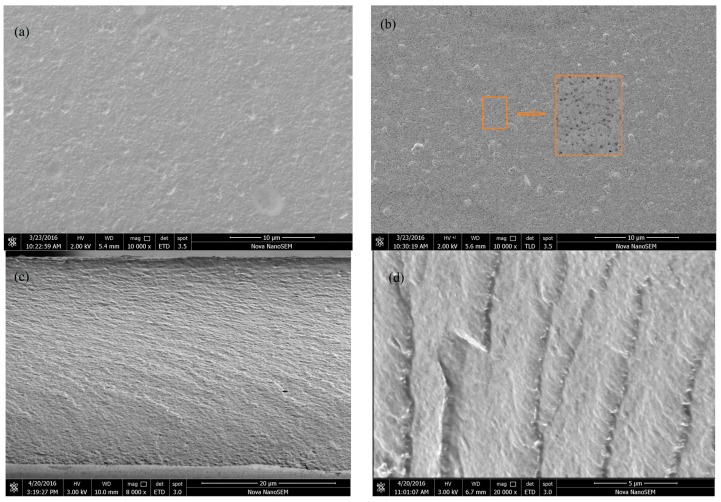
(**a**) SEM of BC/CSMs surface; (**b**) SEM of BC/CS-NFMs surface; (**c**) SEM of BC/CSMs cross-section; and (**d**) SEM of BC/CS-NFMs cross-section.

The surface and cross-sectional microstructure of BC/CSMs and BC/CS-NFMs were characterized by FE-SEM (Figure 6). As observed from Figure 6a, BC/CSMs were smooth and dense. No pore structure could be found on the surface of BC/CSMs. Figure 6c shows that the cross-sectional structure of BC/CSMs were also relatively dense. This is in agreement with the reported results [35]. BC/CSMs were modified to obtain BC/CS-NFMs; Figure 6b shows that obvious pores were observed on the surface, with the pore size range from 30 to 200 nm. The cross-sectional image exhibits a spongy layered structure in BC/CS-NFMs, as shown in Figure 6d. The FE-SEM analysis results indicate that hydrolysis and carboxymethylation have a significant effect on the surface and cross-sectional morphology structure of the BC/CSMs. This behavior may be attributed to strong molecular inter-atomic forces and hydrogen bonding in BC/CSMs; after modification, the hydrogen bonding was damaged, which disrupted the crystalline structures of the cellulose and chitosan, and therefore, the internal structure became loose and an enhanced surface hydrophobicity was observed. Therefore, after modification, BC/CSMs have the function of nanofiltration.

### 3.5. FT-IR Analysis of Cellulose/Chitosan Nanofiltration Membranes

**Figure 7:**
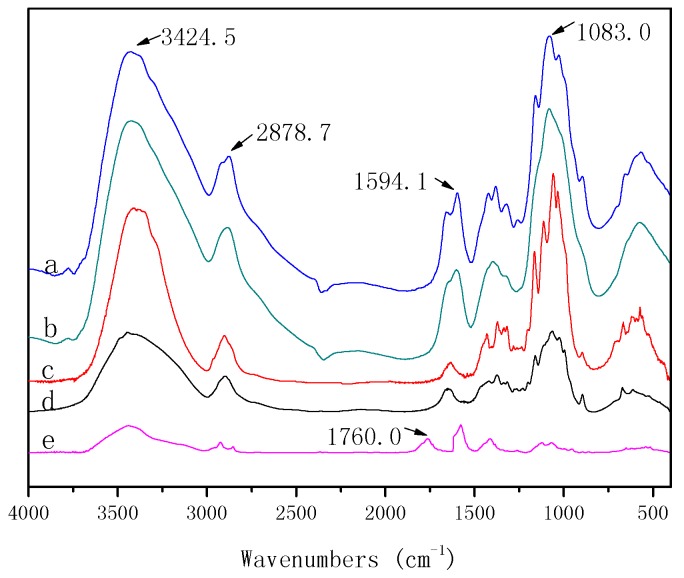
FTIR spectra of CS (**a**); BC/CSMs (**b**); BCM (**c**); BC (**d**); and BC/CS-NFMs (**e**).

The results of the FT-IR spectra of BC, CS, BCM, BC/CSMs, and BC/CS-NFMs are presented in Figure 7. By comparing the spectra and data of BC, CS, BCM, and BC/CSMs, it was observed that the four spectra are similar, which indicates that a chemical reaction does not occur in the process of dissolution and in the coagulation bath during the preparation of BC/CSMs, and only physical changes occur during the process. The results were similar to earlier results observed in the blends of chitosan and cellulose using ZnCl_2_·3H_2_O as the solvent [36]. Strong peaks at 1594.1, 1597, and 1577.6 cm^−1^ arise from the –NH bending amide II, which is present in the chitosan structure. The –NH bending amide II of chitosan makes a greater contribution to the antibacterial activity. The peak at 1760 cm^−1^ arises from the C=O of carboxyl, which is present in BC/CS-NFMs; this shows that a part of hydroxyl in cellulose transforms to carboxymethyl during the hydrolysis and carboxymethylation of BC/CSMs.

### 3.6. X-ray Diffraction of Cellulose/Chitosan Nanofiltration Membranes

**Figure 8:**
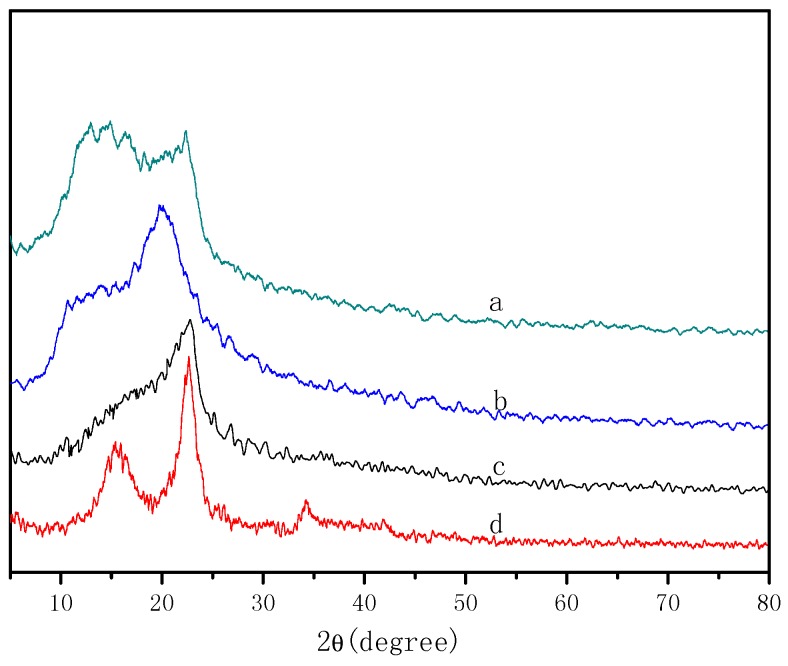
X-ray diffractograms of BC/CS-NFMs (**a**); CS (**b**); BCM (**c**); and BC (**d**).

Figure 8 shows the X-ray diffractograms of BC, CS, BCM, and BC/CS-NFMs. The XRD pattern of BC shows three diffraction peaks at 2θ = 15.4°, 22.6°, and 34.2°, which originate from the cellulose (101), (002), and (040) crystalline planes [37]. However, the diffraction pattern of the BCM exhibits only a peak at 2θ = 22.7°, and no other peaks were observed, which suggests that during the dissolution and regeneration process of cellulose, a transformation of the crystalline structure from cellulose I to cellulose II occurs [38]. In addition, the crystallization degree of BCM was lower than that of BC, but the native crystal structure of BC did not change. The XRD pattern of CS exhibits two diffraction peaks at 2θ = 10.6° and 20.1°, which originate from the chitosan (020) and (100) crystalline planes [39]. The XRD pattern of BC/CS-NFMs show two diffraction peaks at 2θ = 12.9° and 22.3°, indicating that during the BC/CS-NFMs preparation, the crystallinity of BC/CS-NFMs are between the corresponding values of BC and CS. This result may be attributed to the reformation of hydrogen bonds between BC and CS during the dissolution and regeneration processes.

### 3.7. The Permeation and Rejection of Cellulose/Chitosan Nanofiltration Membranes 

Before the modification of the dense BC/CSMs, there is almost no water flux. The filtration performance of the blend nanofiltration membranes (BC/CS = 4:1, 6:1, 8:1, 10:1) was investigated using five aqueous solutions (NaCl, Na_2_SO_4_, MgSO_4_, methyl orange, and methyl blue). The water flux and rejection rate results are shown in Table 2. The results indicate that a larger water flux and a higher rejection rate are obtained in aqueous dye-salt solutions through hydrolysis and carboxymethylation. The water flux and rejection rate of different weight proportions of nanofiltration membranes exhibit no obvious changes. It was also found that the rejection rate of divalent ions were higher than that of monovalent ions, and the rejection rate of large-molecular-weight species was higher than that of small-molecular-weight species. After hydrolysis and carboxymethylation, the improved nanofiltration performance is attributed to both the loose internal structure and the enhanced surface hydrophobicity, which is in agreement with the morphology analysis. The divalent cation rejection experiment results showed that the rejection was mainly attributed to size exclusion. With the polyethyleneimine/sulfonation of the polyethersulphone (PEI/SPES) composite nanofiltration membrane [40], under the operating pressure of 0.4 MPa at room temperature, and a water flux of the PEI/SPES composite nanofiltration membrane at 5.8 L/m^2^·h, the rejections to Na_2_SO_4_ and NaCl were 29% and 18%, respectively. Compared to the PEI/SPES composite nanofiltration membrane, BC/CS-NFMs demonstrated better permeation and rejection.

### 3.8. Molecular Weight Cut-Off and Mean Pore Size of BC/CS-NFMs

The MWCO and pore size of the BC/CS-NFMs were determined through PEG retention tests. In this study, PEG was selected because of its low interaction with the membrane material [41]. The corresponding MWCO was obtained from the rejection of different single-solutions of PEG versus their molecular weights, and the Stokes radius of BC/CS-NFMs were calculated (Table 3). From the results, different proportions of cellulose and chitosan exhibit no obvious effect on the MWCO, which corresponds with the water flux and rejection rate analysis. Therefore, the BC/CS-NFMs reached the nanofiltration level.

Therefore, BC/CS-NFMs showed good nanofiltration performance in desalinating salts and removing dyes, and it has a considerable application potential. It is suggested that BC/CS-NFMs could be applied in drinking water purification, seawater desalination, wastewater treatment, and more. Therefore, it is possible that biodegradable membranes can replace non-biodegradable petrochemical membranes in the future.

## 4. Conclusions

In this work, BC/CS-NFMs were successfully prepared from cellulose and chitosan using NMMO as a solvent. The following conclusions can be drawn. BC/CS-NFMs were prepared through the hydrolysis and carboxymethylation of BC/CSMs. The effects of the blend membrane properties are well characterized by determining the mechanical strength, antimicrobial activity, salts and dyes filtration performance, and PEG retention using TGA, FE-SEM, FT-IR, and XRD. The mechanical strength properties of the cellulose and chitosan blend membranes are mainly dominated by cellulose. The strong antimicrobial activity against *Escherichia coli* shows that the antibacterial ability of chitosan was not destroyed. The chemical and physical structures of the blend nanofiltration membranes were modified by hydrolysis and carboxymethylation. The BC/CS-NFMs have good performance for nanofiltration, and the rejection rates of NaCl, Na_2_SO_4_, and MgSO_4_ were more than 30%, 65%, and 65%, respectively. The rejection rates of methyl orange and methyl blue were more than 90%. The pore size of the BC/CS-NFMs were less than 1 nm. The biodegradable, inexpensive, and good separation performance of the nanofiltration membranes will be widely used in water treatment, biotechnology, the food industry, and gas separations.

## Figures and Tables

**Table 1:** Characteristic values of TG and DTG curves.

Samples	*T*_onset_ (°C)	*T*_maximum rate_ (°C)	Residual quantity (%)
BC	303.0	347.9	6.58
CS	245.4	293.3	39.91
BC/CS = 4:1	269.3	333.0	27.72
BC/CS = 6:1	263.1	335.5	25.30
BC/CS = 8:1	257.2	338.0	23.77
BC/CS = 10:1	255.4	335.4	20.55

**Table 2:** Salt and dye filtration performance of BC/CS-NFMs.

BC/CS-NFMs	NaCl ^b^ aqueous solution		Na_2_SO_4_^b^ aqueous solution		MgSO_4_^b^ aqueous solution		Methyl orange ^b^ aqueous solution		Methyl blue ^b^ aqueous solution	
	Water flux ^a^ (L/m^2^·h)	Rejection rate (%)	Water flux ^a^ (L/m^2^·h)	Rejection rate (%)	Water flux ^a^ (L/m^2^·h)	Rejection rate (%)	Water flux ^a^ (L/m^2^·h)	Rejection rate (%)	Water flux ^a^ (L/m^2^·h)	Rejection rate (%)
BC/CS = 4:1	13.63 ± 0.13	34.21 ± 0.42	12.27 ± 0.15	67.58 ± 0.57	12.56 ± 0.14	66.29 ± 0.43	13.76 ± 0.14	92.19 ± 0.35	12.50 ± 0.14	98.68 ± 0.56
BC/CS = 6:1	13.51 ± 0.16	34.42 ± 0.56	12.12 ± 0.17	67.71 ± 0.64	12.43 ± 0.16	66.83 ± 0.56	13.64 ± 0.17	92.37 ± 0.45	12.37 ± 0.15	98.79 ± 0.45
BC/CS = 8:1	13.49 ± 0.11	34.53 ± 0.34	12.03 ± 0.13	67.98 ± 0.59	11.87 ± 0.12	67.12 ± 0.48	13.59 ± 0.18	92.46 ± 0.38	12.23 ± 0.13	98.81 ± 0.62
BC/CS = 10:1	13.21 ± 0.12	34.87 ± 0.44	11.78 ± 0.14	68.23 ± 0.55	11.66 ± 0.15	67.56 ± 0.47	13.26 ± 0.15	92.68 ± 0.44	12.19 ± 0.14	98.83 ± 0.47
BC	13.12 ± 0.15	34.93 ± 0.58	10.32 ± 0.19	68.42 ± 0.52	11.24 ± 0.13	67.95 ± 0.44	12.31 ± 0.12	93.02 ± 0.37	10.12 ± 0.16	98.91 ± 0.48

^a^ Tested with a salt or dye aqueous solution under 0.5 MPa at room temperature; ^b^ Tested with de-ionized water containing 500 mg/L salt or 100 mg/L dye under 0.5 MPa at room temperature.

**Table 3:** Molecular weight cut-off (MWCO) and mean pore size (*r*) for BC/CS-NFMs under different proportions of cellulose and chitosan.

BC/CS-NFMs	Molecular weight cut-off ^a^, MWCO (Da)	Mean pore size, *r* (nm)
BC/CS = 4:1	785	0.68
BC/CS = 6:1	716	0.65
BC/CS = 8:1	702	0.64
BC/CS = 10:1	689	0.62

^a^ MWCO determined using PEG solutions. Tested with de-ionized water containing 100 mg/L PEG solutions under 0.5 MPa at room temperature.
